# Individualized Target Fortification of Breast Milk: Optimizing Macronutrient Content Using Different Fortifiers and Approaches

**DOI:** 10.3389/fnut.2021.652641

**Published:** 2021-09-21

**Authors:** Stephanie Fusch, Gerhard Fusch, Efrah I. Yousuf, Markus Rochow, Hon Yiu So, Christoph Fusch, Niels Rochow

**Affiliations:** ^1^Department of Pediatrics, University Medicine Greifswald, Greifswald, Germany; ^2^Department of Pediatrics, Kantonsspital Aarau AG, Aarau, Switzerland; ^3^Department of Pediatrics, McMaster University, Hamilton, ON, Canada; ^4^Carl-Thiem-Hospital, Cottbus, Germany; ^5^Department of Mathematics and Statistics, Oakland University, Rochester, MI, United States; ^6^Department of Pediatrics, General Hospital, Paracelsus Medical University, Nuremberg, Germany; ^7^Department of Pediatrics, Rostock University Medical Center, Rostock, Germany

**Keywords:** human milk, milk analysis, standard fortification, fat, carbohydrates, protein, preterm infants, growth

## Abstract

**Background:** Native breast milk composition displays significant inter- and intra-individual variation which persists after standard fortification with fixed doses and challenges target fortification. This study aims to analyze the macronutrient composition of different commercially available fortifiers and the effect of different fortification strategies on nutritional intake of preterm infants.

**Methods:** In 103 preterm infants, native breast milk samples were collected from 24-h feeding batches (*n* = 3,338) and fat, protein and carbohydrate contents were analyzed. Nutrient content was compared for breast milk that had undergone either (i) standard fortification, (ii) targeted fortification, (iii) selective batching according to breast milk composition, or (iv) partial lyophilization. For (i) eight commercially available standard fortifiers were tested. Targeted fortification (ii) involved the addition of single component modulars of either protein, fat or carbohydrates to standard fortified breast milk. Using a mathematical growth model, the combined effect of protein, fat and carbohydrate intake on growth was assessed. The best composition of standard fortifiers as the initial step for target fortification was explored assuming three clinical scenarios for milk analysis.

**Results:** Macronutrient content was highly variable between native breast milk samples, and this variation was still present after standard fortification, however at elevated macronutrient levels. Standard fortification, breast milk batching, as well as partial lyophilization of human milk resulted in deficient and imbalanced enteral intakes in a significant proportion of infants. Target fortification reduced this variation in a, respectively, higher percentage of samples. The effect size was dependent on the number of measurements per week. The optimum composition of standard fortifiers was dependent on the clinical scenario (measurement frequency) for target fortification.

**Conclusions:** To provide precise and accurate intakes of macronutrients, breast milk should be target fortified. Standard fortified breast milk can result in excess above recommended intakes of some macronutrients which limits the efficiency of target fortification. Standard fortifiers with improved composition are needed for target fortification.

## Introduction

**Figure 1:**
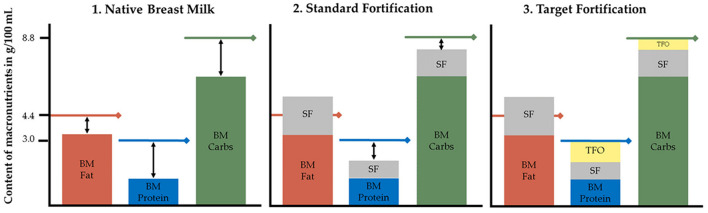
Macronutrient content in native breast milk, standard fortified and target fortified breast milk. Lines represent recommended macronutrient contents of 4.4 g fat, 8.8 g carbs, and 3.0 g protein per 100 mL to reach ESPGHAN recommended intakes assuming an average fluid intake of 150 mL/kg/d leading to a total daily intake of 6.6, 13.2, and 4.5 g/kg/d for fat, carbs, and protein, respectively. BM, native breast milk; Carbs, carbohydrates; SF, standard fortification; TFO, target fortification. Under certain conditions single nutrient levels can exceed ESPGHAN recommendations already after standard fortification.

Between 43 and 97% of preterm infants experience postnatal growth restriction (1, 2). Adequate nutrition of preterm infants is known to improve neurodevelopmental outcomes in later life and to reduce the risk of chronic cardiovascular and metabolic diseases in adulthood (3–8). For term infants, breast milk is considered as the optimal source of nutrition. It provides macronutrients such as proteins, fat, carbohydrates (carbs), as well as micronutrients that include vitamins and minerals. Breast milk is also known to have positive immunomodulatory and psychological effects and reduces the risk of developing many diseases like respiratory tract infections and allergic diseases (9, 10). Preterm infants fed breast milk during hospitalization in neonatal intensive care units (NICUs) have a lower risk of developing infections and necrotizing enterocolitis (4, 11–18). However, for preterm infants to reach intrauterine growth rates (19), in general higher amounts of nutrients are needed, which cannot be obtained solely from native breast milk [Figure 1] (1).

Fortification of native breast milk is routinely used to meet clinical recommendations of enteral nutrient intake (20). Various types of fortification methods are used to increase the macronutrient content of breast milk. Standard fortification assumes an average composition of breast milk and aims to improve nutrient intake by adding a fixed dose of fortifier to native breast milk (Figure 1). However, because native breast milk shows significant inter- and intraindividual variation of nutrient contents, this fortification strategy is frequently leading to an unbalanced intake (21–23). This will result in suboptimal growth, but in clinical routine it usually will take a few days to recognize and adjust intake—valuable time that is lost for appropriate growth. Adjustable fortification is an alternative approach originally reported to adjust protein intake based on blood urea nitrogen levels. However, the adjustment of deficient nutrients is delayed because it reacts on dynamics of metabolic response to an inappropriate supply (24, 25). Conversely, target fortification aims to provide infants with fortified breast milk that adheres to current clinical recommendations for macronutrient intake. Based on actual measurements of macronutrient contents in native breast milk, one-component modulars of either protein, fat or carbs are added to standard fortified breast milk to reach targeted macronutrient levels (Figure 1). While this process is the most accurate and allows infants to be fed the prescribed dose of macronutrients, it is also the most time-consuming method (26–28).

In the current paper, we examine differences in macronutrient contents of native breast milk samples and the impact of standard and targeted fortification on this variation when using different standard fortifiers and analysis schedules. Further, in this study we aimed (1) to identify the best composition of a standard fortifier for three clinical scenarios for target fortification; (2) to analyze the effect of fortification on infant's growth based on established growth models; and (3) to study pre-fortification approaches to optimize the macronutrient content by batching and lyophilization.

## Materials and Methods

### Study Design and Study Population

**Figure 2:**
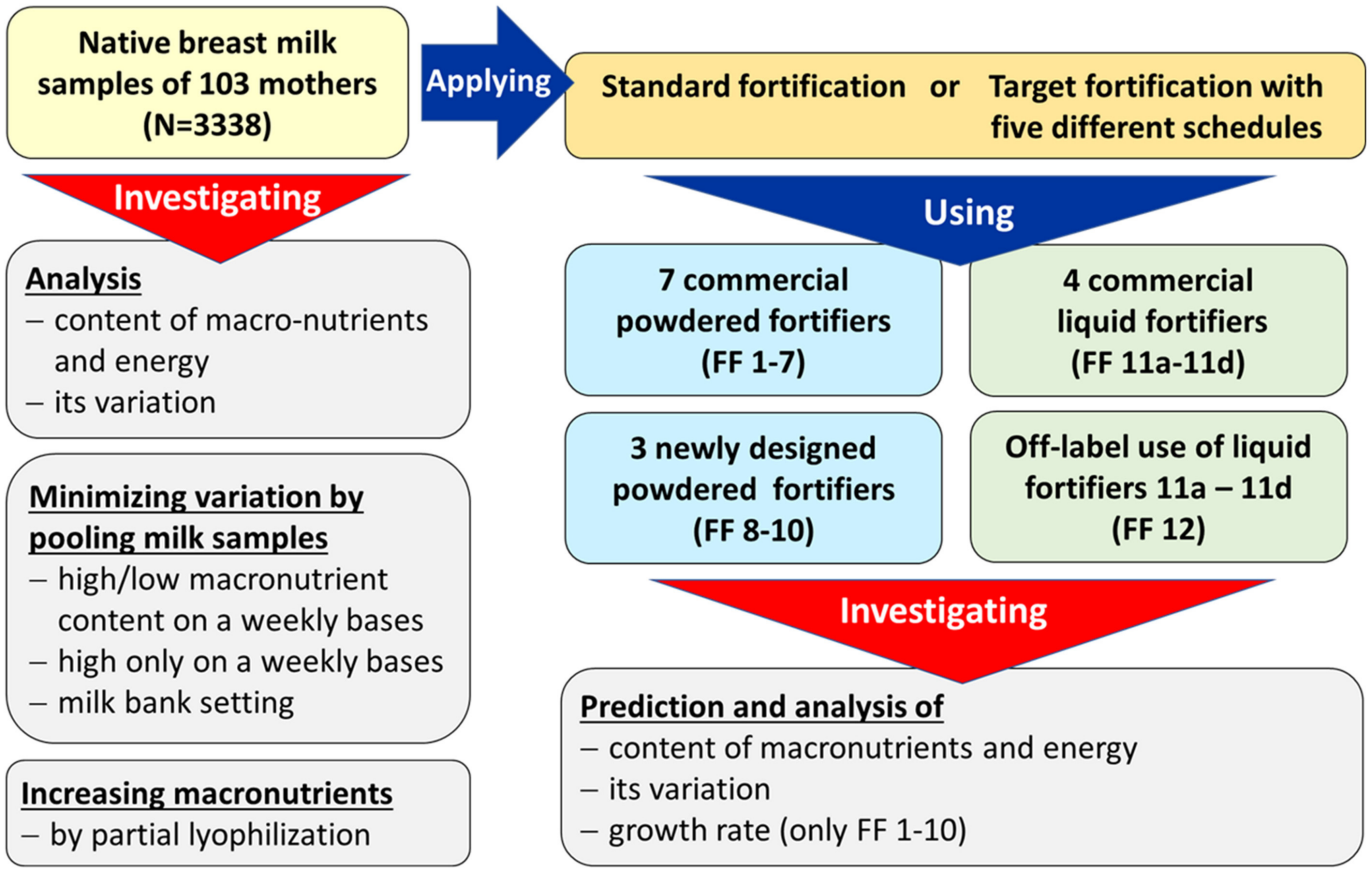
Workflow of experiments (FF, fortifier).

This modeling study is a secondary analysis of a dataset obtained during a randomized controlled clinical trial (RCT) which was conducted between November 2012 and July 2016 at the Division of Neonatology (Level-III NICU) at McMaster Children's Hospital (Hamilton, Ontario, Canada) (28). The purpose of this RCT was to study the effect of target fortification of breast milk on growth, metabolism and neurodevelopmental outcomes using three single macronutrient modular products and compare it to standard fortification. The study was approved by Research Ethics Board of McMaster University (#12-109). All parents gave informed written consent prior to inclusion into the study. The flowchart of performed experiments is outlined in Figure 2.

### Sample Collection and Breast Milk

Samples were collected from pooled batches (*n* = 3,338) of 24-h feedings before breast milk was fortified. Batches were usually prepared from frozen milk of the own mother (MOM) and—depending on the level of available MOM—supplemented with pasteurized frozen human donor milk. Data from 103 preterm infants born at a mean gestational age of 27.0 ± 1.5 weeks (birth weight 980 ± 240 g, head circumference 25.1 ± 2.7 cm, and length 34.7 ± 3.3 cm) were available. Fortification (and subsequent collection of breast milk samples) began at day of life 21 ± 6 and continued over an average period of 33 days ± 10 days. Each study participant provided samples covering at least 14 consecutive days. The native breast milk samples were stored at −20°C until milk analysis.

Breast milk samples were analyzed using validated methods. Samples were thawed and homogenized using a sonicator for 15 s (VCX 130; Chemical Instruments AB, Sollentuna, Sweden). Content of true protein and fat was determined by a validated near-infrared milk analyzer (SpectraStar; Unity Scientific, Brookfield, Connecticut) (29, 30). Lactose was measured using an established reference method (UPLC-MS/MS) in order to avoid crossover from non-digestible carbohydrates, i.e., human oligosaccharides (31).

### Descriptive Analysis of Breast Milk Content and Macronutrient Targets After Fortification

Levels of fat, protein, carbohydrate, and lactose [g/100 mL] were measured in all native breast milk samples. The energy content (kcal/100 mL) was calculated using energy equivalents which assume that protein and carbs yield 4 kcal/g and that fat yields 9 kcal/g of energy, respectively (Atwater factor) (32). Additionally, the protein-to-energy ratio (P:E ratio, [g protein/100 kcal]) (33) and the ratio of total carbohydrate energy vs. non-protein energy was calculated (carbs/NPE, %) (34) for native breast milk as well as for all fortified variations.

Target macronutrient intake for preterm infants <1,000 g of birth weight were defined according to the guidelines of the European Society for Pediatric Gastroenterology Hepatology and Nutrition (ESPGHAN): 4.5 g/kg/d protein, 6.6 g/kg/d fat, and 13.2 g/kg/d carbs (20). With an assumed daily fluid intake of 150 mL/kg/d, nutrient contents of enteral feeds then translate to 3 g of protein, 4.4 g of fat, 8.8 g of carbohydrates per 100 mL of feeds. Achieving the recommended amounts of macronutrients would result in 87 kcal/100 mL, a protein -to-energy (P:E) ratio of 3.5 g/100 kcal and carbohydrate-to-NPE ratio of 47%.

### Standard Fortifier

In this study, seven commercially available multicomponent powdered standard fortifiers (fortifiers 1–7) with different compositions were used. Each of them provides 1.0–1.5 g of extra protein per 100 ml thereby comprising comparable energy intakes. Based on their non-protein composition, they can be divided into three groups: (i) low-fat/high-carbohydrate, (ii) medium-fat/medium-carbohydrate, and (iii) high-fat/low-carbohydrate content (Table 1). For each 24 h-feeding, final macronutrient levels, energy content and corresponding ratios were calculated for all fortifiers.

**Table 1:** Macronutrients added at recommended doses of fortifiers to obtain 100 mL of ready-to-feed breast milk, ^*^g per portion, ^$^kcal per portion (carbs, carbohydrates; LF, liquid fortifier).

**Fortifier group**	**Standard multicomponent powdered fortifie**										**Liquid fortifier based on human milk**			
	**Low-fat/high-carbs**		**Medium-fat/medium-carbs**			**High-fat/low-carbs**		**Optimized standard fortifier**						
**Fortifier #**	**1**	**2**	**3**	**4**	**5**	**6**	**7**	**8**	**9**	**10**	**11a**	**11b**	**11c**	**11d**
Ratio of LF: breast milk	–	–	–	–	–	–	–	–	–	–	20:80	30:70	40:60	50:50
Fat (g/100 mL)	0	0.02	0.72	0.36	0.7	0.7	1	0.77	0	0	1.8^*^	2.7^*^	3.6^*^	4.5^*^
Protein (g/100 mL)	1.1	1	1.4	1	1.3	1.5	1.1	1.85	1.54	1.26	1.2^*^	1.8^*^	2.4^*^	3^*^
Carbohydrates (g/100 mL)	2.7	3.3	1.3	1.8	1.5	0.01	0.4	2.13	0.99	0.45	1.8^*^	2.8^*^	3.6^*^	4.6^*^
Energy (kcal/100 mL)	15	18	17	14	17	13	14	23	10	7	28^$^	42^$^	56^$^	71^$^

In a second step, the optimum macronutrient composition for standard fortification was identified based on three different scenarios for milk analysis as described below (fortifiers 8–10). Composition was optimized for each macronutrient to minimize the deviation between fortified breast milk content and ESPGHAN recommended intake by minimizing the residual sum of squares. In a next step, the performance of fortifiers was evaluated for the three different measurement scenarios. Fortifier 8 represents a fortifier that was optimized for standard fortification only, i.e., no measurement of macronutrients in native breast milk. Fortifiers 9 and 10 were optimized for target fortification using either one breast milk analysis per week (occurring on Monday) or three breast milk analyses per week (occurring on Monday, Wednesday, Friday), respectively.

Further, the macronutrient intake for human milk based liquid fortifiers (fortifiers 11a-d and 12) were also calculated. Fortifiers 11a to 11d represent the different preparation strengths (20–50 mL). Fortifiers 11 and 12 represent the same product, however, fortifier 12 uses different volumes as an off-label clinical approach.

### Target Fortification

Target fortification was calculated using three steps (Figure 1). First, macronutrient contents of native breast milk samples were analyzed (methods see paragraph Sample Collection and Breast Milk). In a second step, a standard fortifier (as described below) was added to native 24-h breast milk batches. Third, based on the measurement of the macronutrient content of breast milk, single component modulars containing either protein, fat and/or carbohydrates were added to standard fortified breast milk to achieve the recommended enteral macronutrient intake. The amount of required modulars was calculated by subtracting the standard fortified breast milk content for fat, protein, and carbs from the ESPGHAN recommended targets (20, 26). Further details and a model calculation have been previously described (28). In cases where the amount of a macronutrient already exceeded the recommendations after standard fortification, this macronutrient was not further fortified; the others were adjusted through the addition of modular products.

### Schedules for Target Fortification

For the target fortification approach, we tested five different schedules for milk analysis and simulated the resulting macronutrient concentrations using fortifiers 1–12 (Table 2).

**Table 2:** Different schedules of target fortification analysis and recipe adjustment (BM—pooled native breast milk batch for 24 h feeding).

**Approach**		**Monday** **BM batch**	**Tuesday** **BM batch**	**Wednesday** **BM batch**	**Thursday** **BM batch**	**Friday** **BM batch**	**Saturday** **BM batch**	**Sunday** **BM batch**
1/week	Analysis	X						
	Recipe	Mo	Mo	Mo	Mo	Mo	Mo	Mo
2/week	Analysis	X			X			
	Recipe	Mo	Mo	Mo	Th	Th	Th	Th
3/week	Analysis	X		X		X		
	Recipe	Mo	Mo	We	We	Fr	Fr	Fr
5/week	Analysis	X	X	X	X	X		
	Recipe	Mo	Tu	We	Th	Fr	Fr	Fr
7/week	Analysis	X	X	X	X	X	X	X
	Recipe	Mo	Tu	We	Th	Fr	Sa	Su

*“X” indicates the day of milk analysis. Same colors indicate the same recipe for target fortification*.

### Target Fortification With Liquid Fortifier

Macronutrient intakes achieved by using commercially available human milk based liquid fortifiers were also investigated. Different from powdered fortifiers, all liquid fortifiers inherently will replace a specific volume of native breast milk. For example, for a final feeding volume of 100 mL, 20 mL of fortifier product will be added to 80 mL breast milk. To achieve higher strengths of fortification the ratio of fortifier-to-breast milk needs to be increased (from 20:80 to 30:70, 40:60, or 50:50 v/v e.g.). In this study, four different fortifier strengths were studied consisting of the following composition per 100 ml of ready-to-feed-batches: 20 mL (fortifier 11a), 30 mL (fortifier 11b), 40 mL (fortifier 11c), and 50 mL (fortifier 11d). All four different liquid fortifier products contain identical macronutrient concentrations (g/mL). The 10 mL- increase in the volume ratio of fortifier to breast milk leads to an incremental increase in protein by ~0.6 g, fat by ~0.9 g, and carbohydrates by ~1.0 g per 10 mL of fortifier.

The following approaches were applied and referred to as fortifier 11 in the analyses: (1) Standard fortification with liquid fortifier: an average protein content of 1.2 g/100 mL in native breast milk was assumed. Fortifier 11b (30:70 v/v ratio) which increases the protein content by 1.8 g/100 mL was chosen as reference fortifier because it represents the fortifier which is most frequently used in clinical routine. (2) For target fortification: Breast milk analysis was performed and the extra protein content needed to reach 3 g/100 mL was calculated. Based on the analysis, the fortifier (11a to 11d) was chosen that was most appropriate to reach the protein target range of 2.8–3.4 g/100 mL. In the final step, fat and carbohydrate content—if below target—were adjusted by adding single component modular products to meet ESPGHAN recommended intake (20).

### Off-Label Use of Liquid Fortifier for Target Fortification

Switching between the four different strengths of the HM based liquid fortifiers (11a, 11b, 11c, and 11d) will lead to stepwise increase of protein and macronutrients intake. To allow a more continuous increase fortifiers were dosed in an off-label approach to achieve ESPGHAN recommendations. This approach is categorized as fortifier 12.

**Figure 3:**
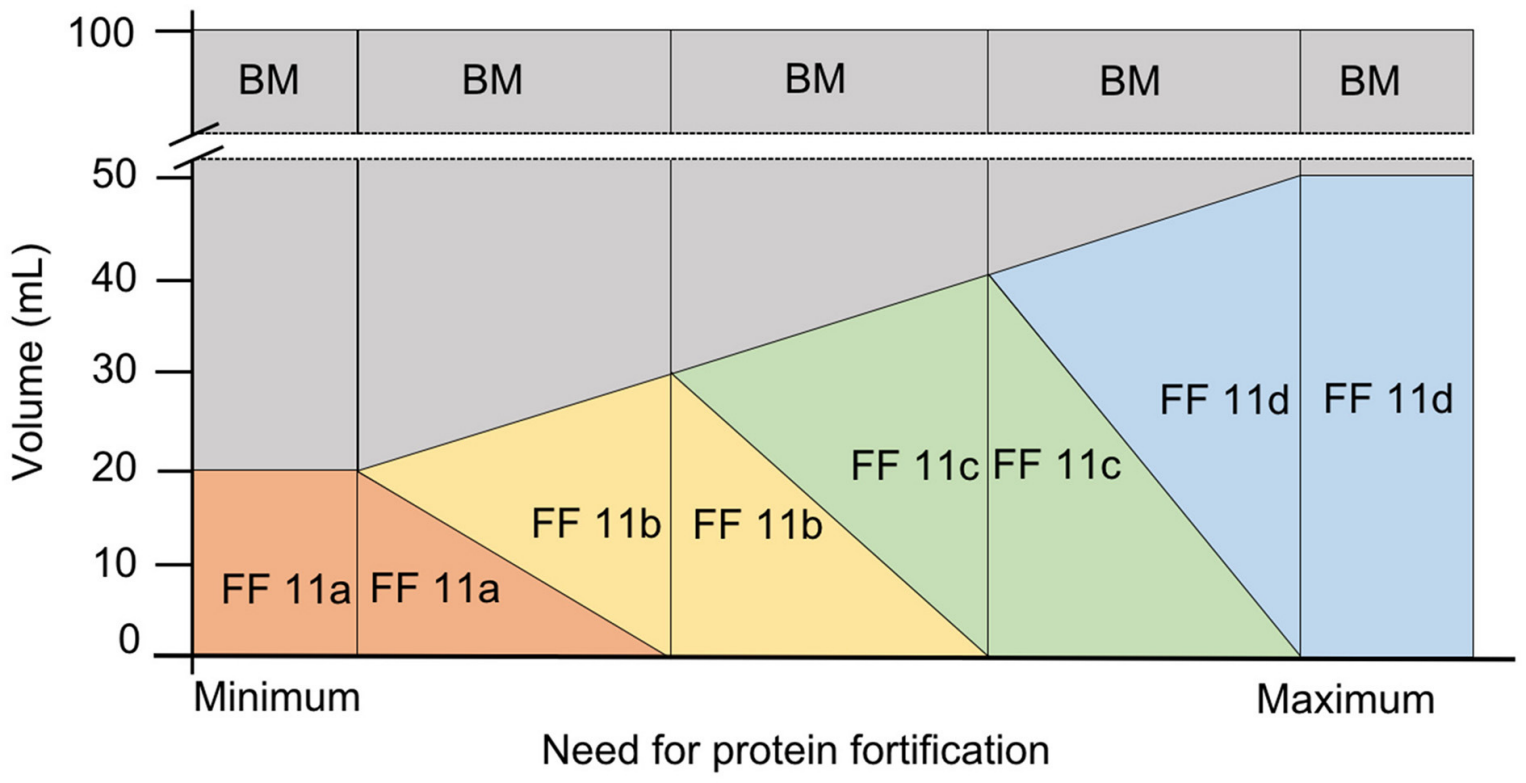
Off-label approach for target fortification with liquid fortifier based on the need of protein fortification in native breast milk (BM, breast milk; FF, fortifier).

For this off-label approach, target fortification was performed by measuring breast milk macronutrient content and native breast milk batches were adjusted to final target protein concentration of 3 g/100 mL. For native breast milk with a protein content of ≥1.8 g/100 mL, liquid fortifier 11a was used as a 20: 80 ratio (i.e., ratio of fortifier: native BM). For native breast milk with a protein content of ≥1.2 to 1.8, the liquid fortifier was 11a was used as the base component and fortifier 11b was added to reach a protein content of 3 g/100 mL. In this case, the ratio between the volume of fortifier 11a and 11b was 2:3 or, as expressed in volume referred to a ready to feed volume of 100 mL, 20 ml of fortifier 11a would be replaced by 15 ml of fortifier 11b, consequently, the volume of native milk needs to be reduced by this amount. For native breast milk with a protein content of ≥0.6 to <1.2 g/100 mL, fortifier 11b constituted the base fortifier and fortifier 11c was added in a ratio of 3:4. For native breast milk with a protein content of ≥0 to <0.6 g/100 mL, fortifier 11c formed the base component and fortifier 11d was added in a ratio of 4:5 (Figure 3).

After target fortification of protein, the final step of adjusting fat to 4.4 g/100 mL and carbs to 8.8 g/100 mL was performed using modular products.

### Identification of Feeds With Insufficient Macronutrient Intake

Because of the natural intra- and interindividual variation of breast milk macronutrient composition standard fortification (i.e., without milk analysis) will always result in over- or under-fortification of a significant number of breast milk samples. To investigate the magnitude of this inherent weakness feeds with inappropriate macronutrient composition shall be identified applying principles of nutritional physiology. Levels of all three macronutrients and energy as well as ratios of protein-to-energy as well as carbohydrate energy-to-non-protein energy were incorporated into an established prediction equation for growth velocity (33–35). Feeds resulting in growth deviating for more than 1 g/kg/d from expected target weight gain velocity would be considered as inappropriate (33–35). To calculate the daily growth of the study infants depending on the nutritional intake routine feeding volumes of 150 mL/kg/d were assumed and the weight gain velocity was calculated per body weight (g/kg body weight per day).

Two nutritional physiological approaches were considered. The base equation was derived from Kashyap et al. (35). This equation considered the effects of protein and energy on growth rates (Equation 1).


(1) $$\begin{array}{l} weight\;gain\;velocity=0.095\cdot energy\;intake+3.6\cdot protein\;\\ \;intake\;-0.00468\cdot BW+1.699 \end{array}$$

with energy intake as [kcal/kg/d], protein intake as [g/kg/d] and birth weight (BW) as (g). For our calculations a constant birth weight of 1,000 g was assumed.

Growth rates are mainly determined by protein and energy intakes, but nutritional studies have shown that variations of the carbohydrate-to-non-protein energy ratio significantly modulate growth rates (33, 34). This impact is quantified by using a metabolic factor given in Equation (2). The equation was obtained from regression analysis performed on previously published growth data of preterm infants fed with same amount of protein and calories, however with different carbohydrate to non-protein energy ratios (33, 34). Combining both equations into one leads to Equation (3) combining the impact of all three macronutrients on weight gain


(2) $$\begin{array}{l} Metabolic\;factor=0.7757+\left(0.47\cdot \frac{Carbs\;energy}{NPE\;energy}\right) \end{array}$$

(3) $$\begin{array}{l} Target\;weight\;gain\;velocity=\left(0.095\;\cdot \;Energy\;intake+3.6\;\cdot \;Protein\;intake-0.00468\;\cdot \;BW+1.699\right)\\ \;.(0.7757+\left(0.47\;\cdot \;\frac{Carbs\;energy}{NPE\;energy}\right)) \end{array}$$

Target weight gain velocity was defined using (Equation 3) thereby assuming ESPGHAN recommended intakes for an infant with a body weight of 1,000 g (4.5 g/150 mL protein, 6.6 g/150 mL fat, and 13.2 g/150 mL carbohydrate), the target weight gain velocity was defined. Macronutrient composition of single feeds was considered as “inadequate” if the estimated weight gain velocity was >1 g/kg/d above or below the target weight.

Human milk based liquid fortifiers were not included in this analysis due to insufficient reference data. Additionally, the fat phase from human milk might have different physiological effects when compared with fat in formula (36).

### Optimization of Macronutrient Content in Native Breast Milk to Reduce the Variation

#### Simulation of Selection and Combination of Specific Native Breast Milk Samples

To investigate whether the natural variation of breast milk can be reduced, pairs of breast milk samples from the same mother with matching macronutrient content shall be pooled. Length of pooling intervals was set to alternating between *n* = 6 and *n* = 8 days (~1 week) to allow paired matching. The interval length was chosen assuming a minimum number of frozen milk samples during the major part of a NICU stay available to prepare feedings. Within each group BM samples were sorted by one macronutrient (either fat, protein, carbs) or by energy content in ascending order. Assuming identical volumes (e.g., 100 mL), as a next step two or three samples were pooled using two different approaches:

(i) *Averaging macronutrient by combining breast milk samples with high and low macronutrient content:* According to the selected macronutrient, pairs of samples (the highest and the lowest value, the second highest and second lowest, proceeding continuously) were pooled. Pooled samples were split into two of equal volume.

(ii) *Combining breast milk samples with the highest macronutrient content:* The three samples containing the highest amounts of the selected macronutrient were mixed and divided into equal samples containing a volume of 100 mL each. The remaining three or five batches were not included into further analysis (see discussion).

The content and variation of fat, protein, carbohydrates, and energy were calculated for each of the resulting batches.

#### Random Milk Pooling to Reduce the Variation of Macronutrients

This simulation addresses the question whether random pooling of native breast milk samples will be able to reduce the variability of macronutrients and, if so, which would be the most efficient set size. For this purpose, a defined number of native breast milk batches were randomly drawn from the original data set of 3,338 batches, were pooled and the resulting content of protein, fat, and carbohydrates was calculated. For any given set size, this step of random allocation was repeated 1,000 times and mean and SD were calculated to serve as an estimate of the most likely distribution of nutrient contents after pooling. Set size started at *n* = 1 and was increased up to *n* = 996 with increments of *n* = 5. Cut-off criterium for efficiency was the minimum set size needed to reduce protein content below 10% variability.

### Optimizing Macronutrient Content by Lyophilization (Freeze-Drying)

This simulation uses partial lyophilization to up-concentrate the breast milk content. Separate analyses were done for protein, fat, and carbohydrates as the leading macronutrient to determine the amount of water to be removed to meet ESPGHAN recommendations at a h feeding volume of 150 mL/kg/d (20). Up-concentrated compositions of the other two macronutrients were calculated using the “new” hydration factor.

### Statistics

Descriptive statistics for the macronutrient content of native breast milk and the different approaches to target fortification were calculated using R version 3.5.3 (2019-03-11, R Development Core Team 2019).

**Figure 4:**
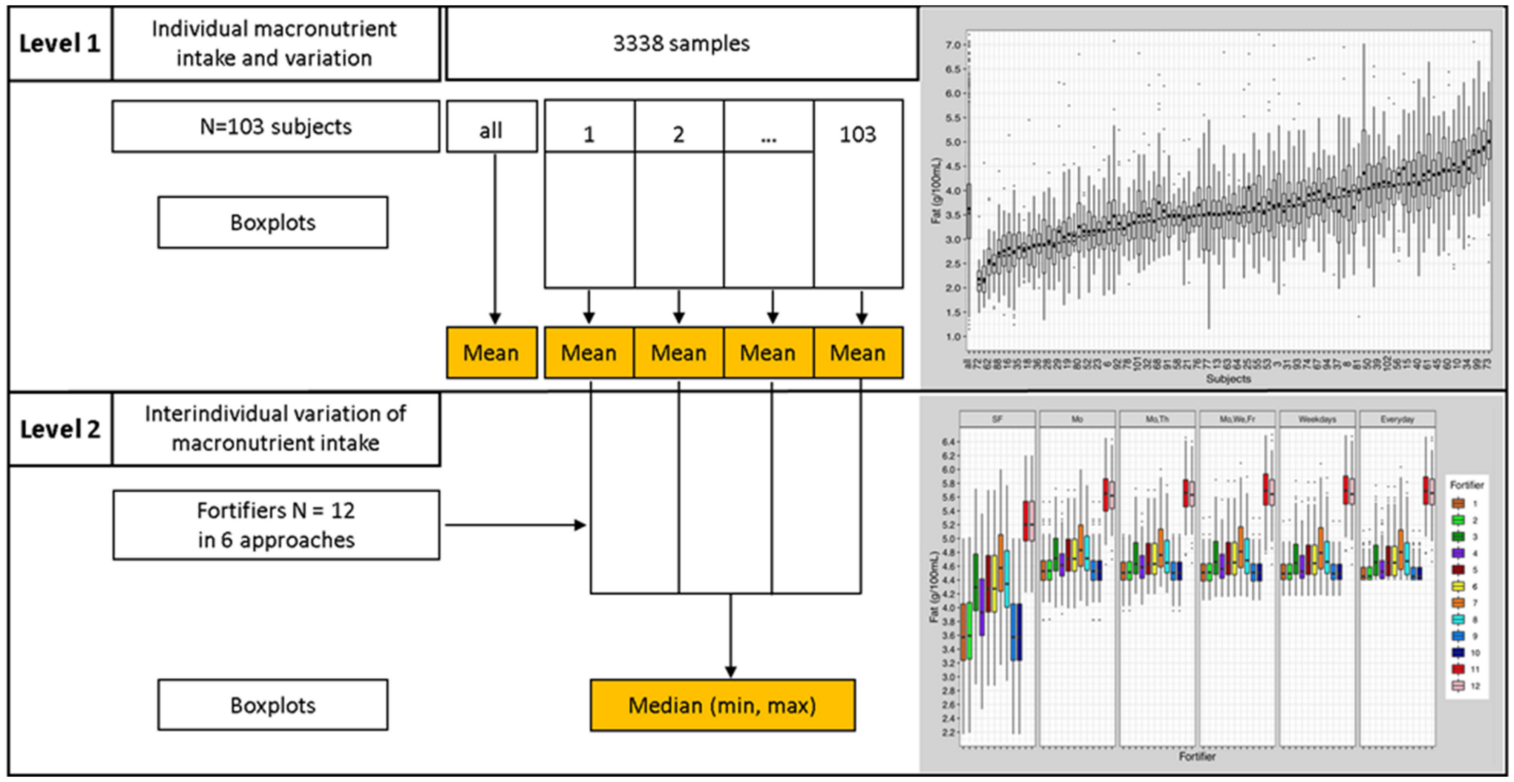
Statistical analyses process; Level 1 analysis—presentation of individual data, Level 2 analysis—inter-individual comparison.

Level 1 analysis analyzed individual data. For each subject (*n* = 103), mean, standard deviation, median, interquartile range, quantile distance Q_0.1_-Q_0.9_, and boxplots were calculated (Figure 4).

Level 2 analysis employed an interindividual analysis. Results from “Level 1 analysis” were used to assess interindividual differences (Figure 4). Level 2 analysis compared and evaluated means and quantile distances from each subject (*n* = 103) of the “Level 1 analysis.” Boxplots and descriptive statistics were generated.

## Results

### Native Breast Milk Analysis and Description

*Overall*, the fat, protein, and lactose content of native breast milk showed high inter- and intra-individual variation and a high range of macronutrient content or composition (Table 3).

**Table 3:** Macronutrient content of *n* = 3,338 native breast milk samples from *n* = 103 subjects.

	**Mean ±*SD***	**Range**
Fat (g/100 mL)	3.6 ± 0.9	1.5–7
Protein (g/100 mL)	1.1 ± 0.3	0.3–2.5
Lactose (g/100 mL)	6.7 ± 0.8	4.0–9.0
Energy (kcal/100 mL)	64 ± 9	40–95
Protein:Energy (g/100 kcal)	1.8 ± 0.4	0.8–3.3
Carbs/NPE (%)	46 ± 7	25–70

*Carbs, carbohydrate; NPE, non-protein energy; Carbs/NPE, carbohydrate energy/carbohydrate energy + fat energy*.

**Figure 5:**
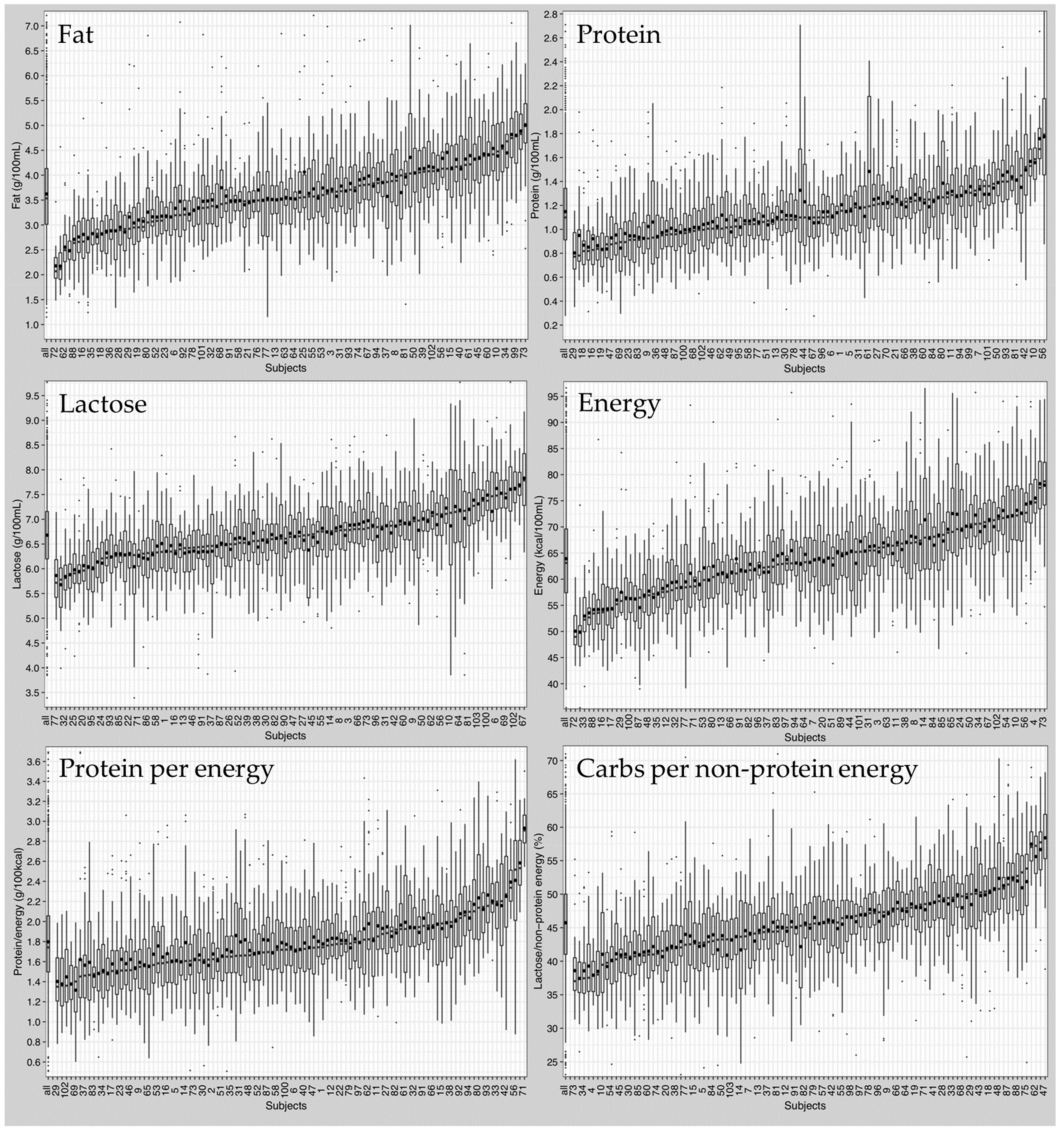
Composition of native breast milk presented in ascending order of median nutrient content per subject. Boxplots display median values of all subjects (left boxplot) and of individual subjects (103 boxplots). Circles represent mean values, dots represent outliers.

*At the intra-individual level*, native breast milk samples from the same mother were also found to vary in macronutrient composition (Figure 5). Boxplots depicting infant macronutrient content were sorted in ascending order of the subject's median fat, protein, and lactose content. The order of subject IDs was found to be different between the three macronutrients (Figure 5). This suggests that fat, protein and lactose content were not correlated with one another. For instance, mothers who had breast milk with high levels of fat did not necessarily have high protein or lactose content in their milk.

In addition to the variation in median macronutrients, the range of macronutrient content was also highly variable between individuals. Independent of the median macronutrient concentration, breast milk samples with low median protein content could have large interquartile ranges, while breast milk samples with high median protein content could show small interquartile ranges or vice versa.

Moreover, the high variation was also observed in calculated parameters like “Protein to Energy Ratio” and “Carbohydrate Energy per Non-Protein Energy” (Supplementary Tables 1, 2).

### Commercially Available Multicomponent Standard Fortifier (Fortifier 1–7 and 11)

**Figure 6:**
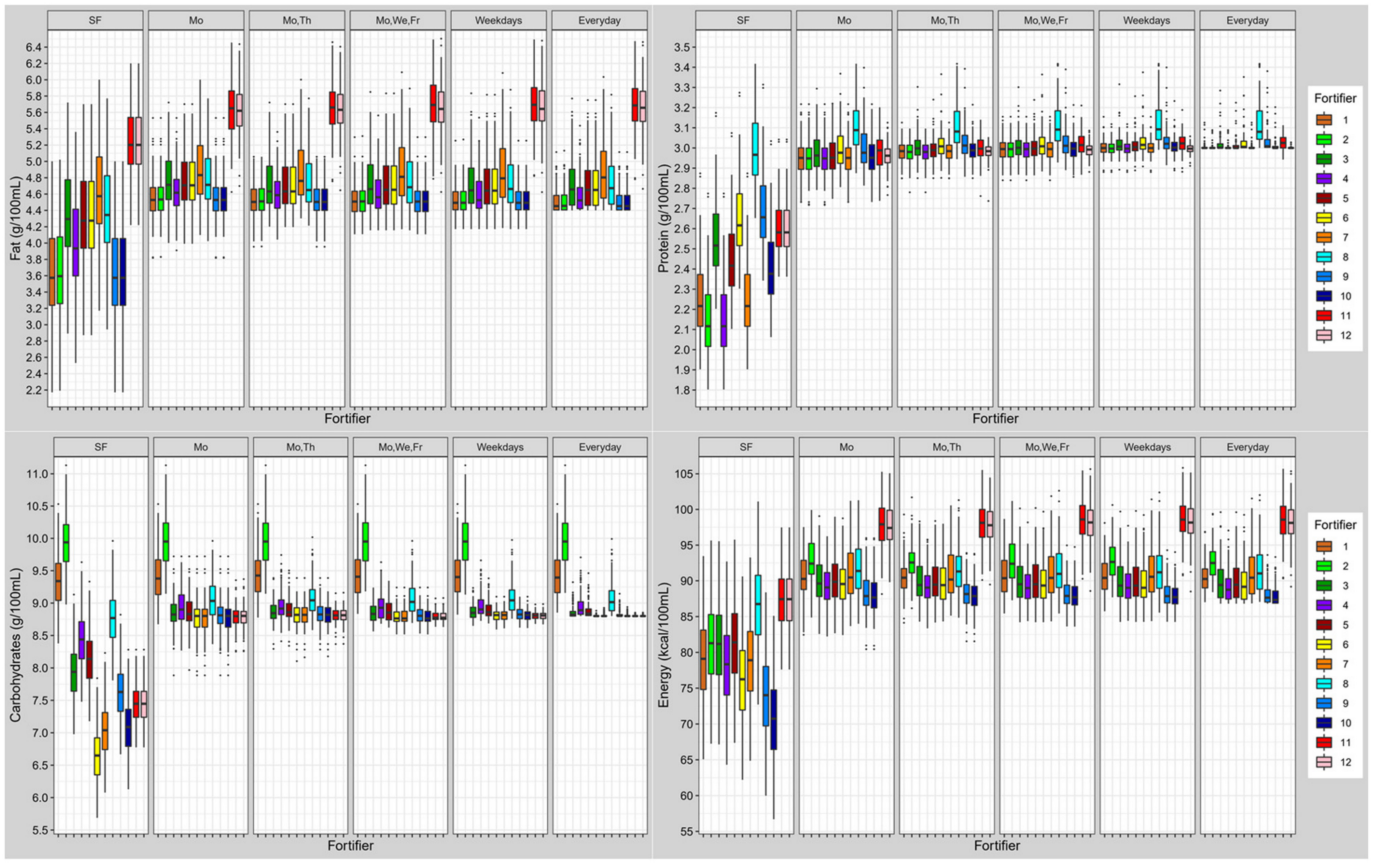
Macronutrient (fat, protein, carbohydrates) and energy content of fortified breast milk with 12 fortifiers using different schedules for milk analysis. Boxplots display the distribution of the mean intake of *n* = 103 subjects. Dots represent outliers (SF, standard fortification; Mo, Monday; We, Wednesday; Th, Thursday; Fr, Friday; SF, fortifier 11 and 12 are identical due to following standard fortification protocol).

**Figure 7:**
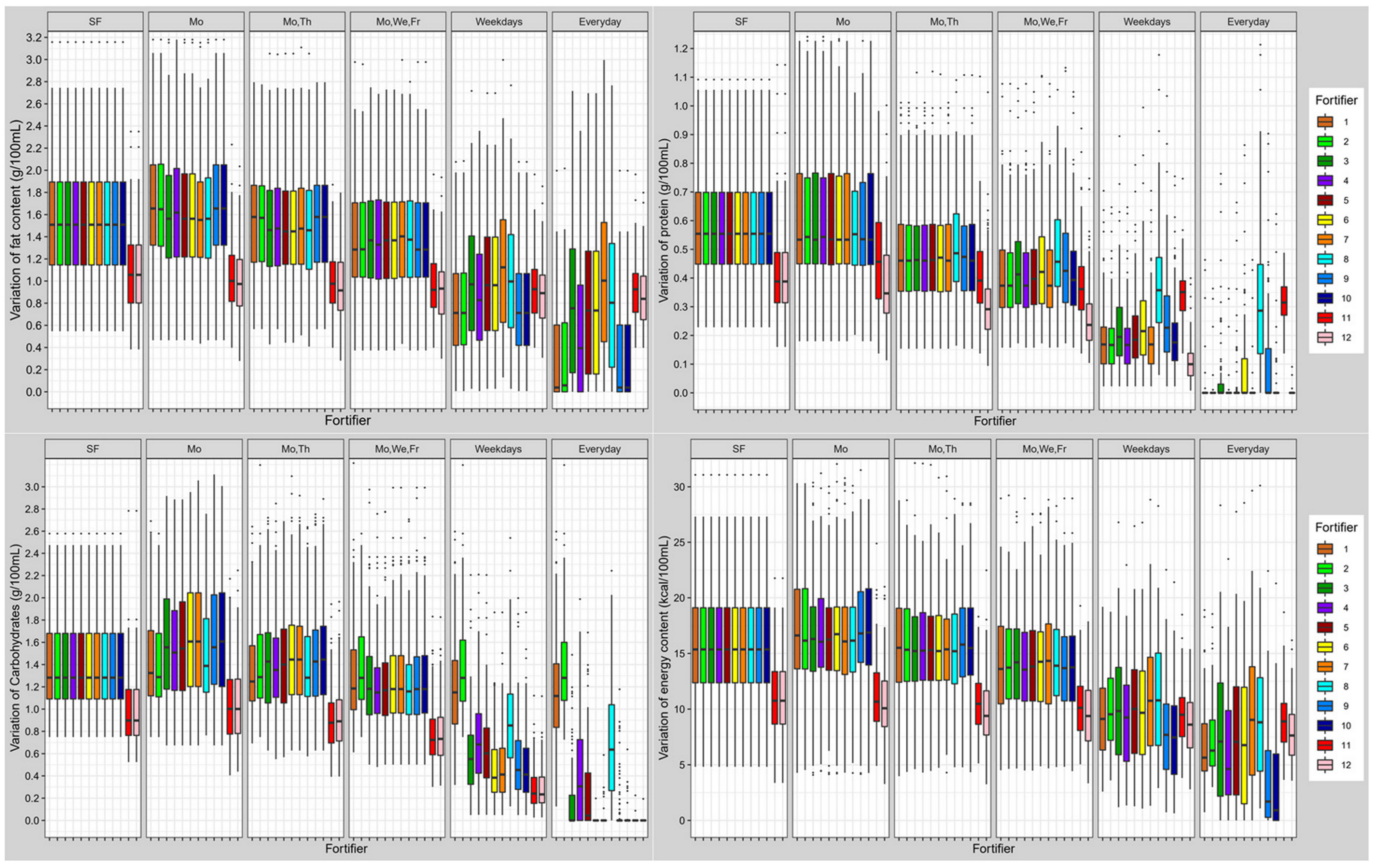
Variation of macronutrient (fat, protein, carbohydrates) and energy content in fortified breast milk using 12 fortifiers with different schedules for milk analysis. Boxplots show the distribution of quantile distances (Q_0.1_-Q_0.9_) of *n* = 103 subjects (SF, standard fortification; Mo, Monday; We, Wednesday; Th, Thursday; Fr, Friday).

*Standard fortified breast milk*. Overall, all commercially available standard fortifiers did not meet recommended nutrient intakes for preterm infants (Figure 6). In most cases, the protein content was below the target (median of 2.1–2.6 g/100 mL instead of 3.0 g/100 mL). For fat and carbohydrate levels results were dependent from the type of fortifier used. For low-fat/high-carbohydrates fortifiers (fortifiers #1 and #2) breast milk batches ended up with median fat levels below 3.6 g/100 mL, and with median carbohydrate levels from 9.3 to 9.9 g/100 mL thus exceeding ESPGHAN recommendations. For medium-fat/medium-carbohydrate fortifiers (fortifiers #3, #4, and #5), median fat and carbohydrate contents were in the range of the recommended intake, however, a significant number of batches still had insufficient carbohydrate content In the high-fat/low-carbohydrates group (fortifiers #6 and #7), median fat content exceeded recommendations (4.3–4.6 g/100 mL), while median carbohydrate content ranged between 6.7 and 7.1 g/100 mL. i.e., below the recommendations (Figure 7). Median energy intake as well as protein to energy ratio (P:E ratio) were also below the targets. The percentage of carbohydrate energy per non-protein energy (Carbs/NPE) was lowest in the high-fat/low-carbs group (fortifiers #6 and #7) (Table 4). Further details are presented in the Supplementary Figures 1, 3.

**Table 4:** Energy ratio of standard fortified breast milk (median) compared to ESPGHAN recommendation achieved with ^*^150 mL/kg/d milk intake, carbs, carbohydrate; P:E ratio, protein to energy ratio; Carbs/NPE, carbohydrate energy per non-protein energy.

	**ESPGHAN targets**	**Standard fortifier by non-protein composition**		
		**Low-fat/high-carbs**	**Medium-fat/medium-carbs**	**High-fat/low-carbs**
Energy (kcal)^*^	130	119–122	117–122	114–119
P:E ratio (g/100 kcal)	3.5	2.6–2.8	2.7–3.2	2.8–3.5
Carbs/NPE (%)	47	54–55	45–49	41

When using the liquid fortifier (#11b), the median fat content exceeded the recommended intake by ~0.8 g/100 mL (Figure 6) while median protein (2.6 g/100 mL) and carbohydrate (7.5 g/100 mL) content were close to ESPGHAN recommendations. However, the median energy content (88 kcal/100 mL) was higher compared to standard powder fortified breast milk (76–81 kcal/100 mL).

Overall, breast milk standard fortified with fortifier 11 still showed a high individual variation of macronutrients. The individual variation (Quantile range Q_0.1_-Q_0.9_) was 0.6 g/100 mL for protein, 1.5 g/100 mL for fat, 1.3 g/100 mL for carbohydrates, and 15 kcal/100 mL for energy. Due to the volume displacement, the use of liquid fortifier (#11b) decreased the variation of macronutrients (protein: 0.4; fat: 1.1; carbohydrates: 0.9 g/100 mL; and energy: 11 kcal/100 mL) when compared to powdered fortifiers (Figure 7).

### Optimized Standard Fortifier (Fortifiers #8, #9, and #10)

Using the macronutrient data obtained from all 3,338 breast milk samples, the optimum composition of a standard fortifier for three given measurement schedules (none vs. once vs. three times per week) was calculated (fortifier 8–10). For all three measurement schedules final macronutrient content of breast milk batches fortified with the corresponding optimized fortifier were then computed (Table 1). Macronutrient composition was found to be different for each of the three optimized fortifiers. Fortifier 8 (standard fortification, no measurements) had the highest macronutrient and energy content (fat 0.8 g, protein 1.9 g, carbs 2.1 g, energy 23 kcal) and on average provided ESPGHAN recommended intakes. However, intra- and interindividual variation of standard fortified breast milk remained unchanged compared to that of native breast milk. As a result, many breast milk batches had macronutrient compositions that were above or below recommendations or had unbalanced ratios, i.e., P:E or carbohydrate-to-non-protein-energy.

Fortifiers 9 and 10 shall serve as base fortifiers for target fortification with either one measurement per week or 3 measurements per week, respectively. Macronutrient levels decreased with increasing measurement frequency. Interestingly, they did not require the addition of fat. Since fat content was highly variable and exceeded recommendations in a significant number of breast milk batches routinely adding more fat seems to be quite unreasonable. As a consequence of this approach, administering any extra fat by single macronutrient modular is not required in ~40% of the feeds. Compared to fortifier 9, fortifier 10 added lower amounts of protein (1.3 vs. 1.5 g) and carbohydrates (0.5 vs. 1.0 g).

### Target Fortified Breast Milk

Overall, target fortification was able to achieve ESPGHAN recommended intakes. Target fortification based on just one breast milk analysis per week provided a median macronutrient intake that met recommended levels. However, some residual day-to-day variation remained. A clinically significant reduction in macronutrient variation was found when target fortification was based on three or more measurements per week (Figure 7).

Target fortification using low-fat/high-carbohydrate standard fortifiers (fortifiers #1 and #2) led to some milk samples exceeding the recommended carbohydrate content without extra modular macronutrients being added, while the use of high-fat/low-carbs fortifiers (fortifiers #6 and #7) resulted in fat levels that exceeded recommendations. In these samples target fortification was not able to reduce the macronutrient variation as well as to achieve recommended intakes. The fortifier optimized for three measurements (fortifiers #10) came the closest to ESPGHAN recommended intakes for three or more measurements. The highest precision and accuracy of nutrient intake was reached with daily measurements.

The use of the liquid fortifier (#11) led to median fat (5.6 g/100 mL) and energy (97 kcal/100 mL) levels that were above the recommended intake. The use of liquid fortifier also reduced the variability of macronutrient content due to volume displacement [see also last paragraph of chapter Commercially Available Multicomponent Standard Fortifier (Fortifier 1–7 and 11)] One measurement per week for target fortification achieved a lower variation when compared with powdered fortifiers.

Detailed data about energy intake, protein per energy ratio as well as carbohydrate energy per non-protein energy are presented in Supplementary Figures 2–4.

### Assessment of Achieved Macronutrient Intake at Standardized Tolerance Range

In a next step, we were aiming to evaluate the quality of nutrient supply provided by the different fortification regimes. For this purpose, rather than assess the quality of each single nutrient intake separately, we focused on the effect of the composite intake on growth. Growth is a function of the intake of all three macronutrients therefore we applied an established growth model to estimate ELBW infant growth rates assuming a fixed intake of 150 mL/kg/d for all fortification approaches (20, 33–35). Growth rates achieved by ESPGHAN recommended intakes were considered to be the reference data.

**Figure 8:**
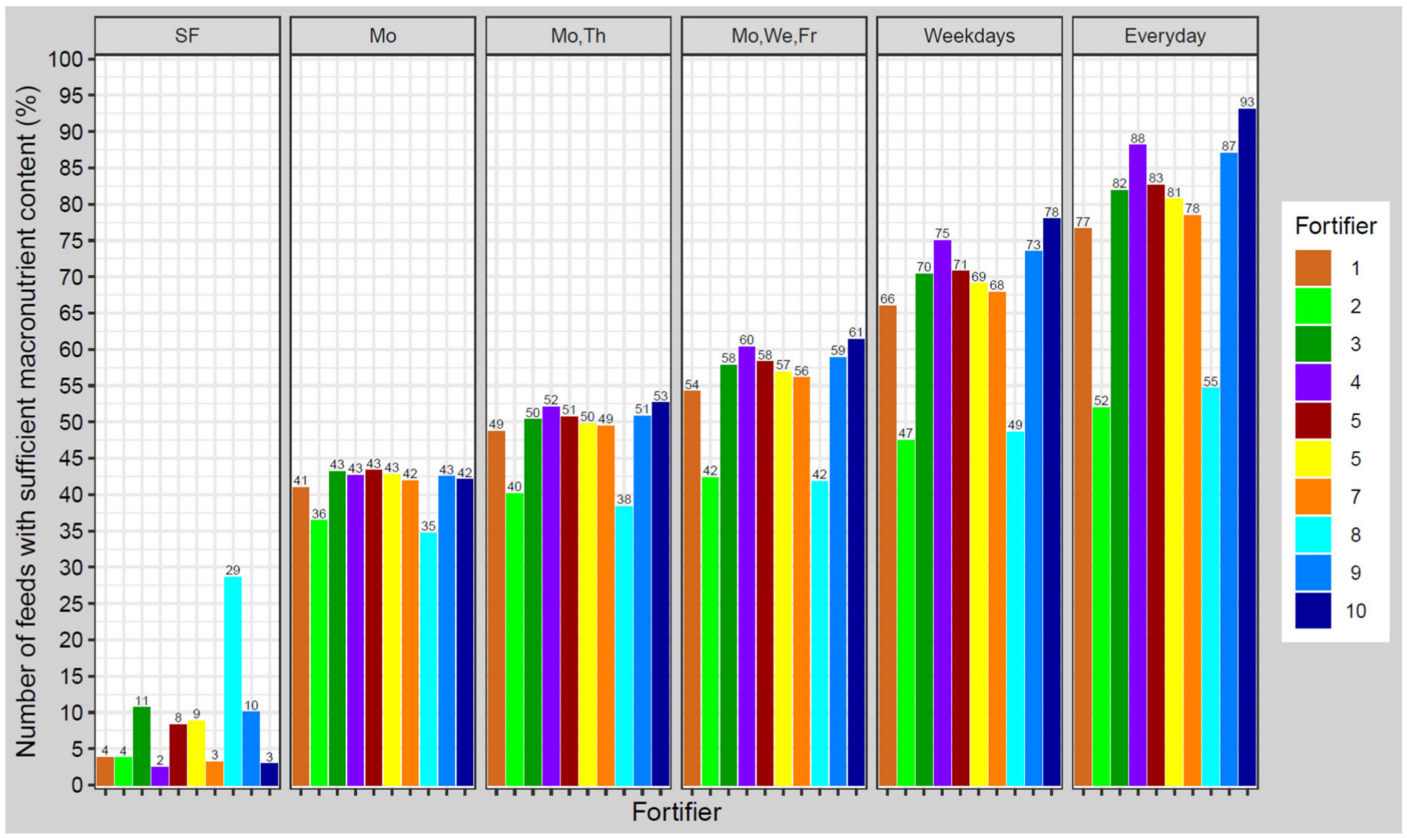
Percentage of feeds with appropriate macronutrient content to achieve growth rates in a range of ±1.0 g/kg/d from estimated target weight gain velocities. Ten different fortifiers in six different scenarios/schedules to measure breast milk macronutrient content were studied (SF, standard fortification; Mo, Monday; We, Wednesday; Th, Thursday; Fr, Friday). The number of fortifiers refers to Table 1.

Applying standard fortification, most samples led to growth rates below the defined tolerance range of ±1 g/kg/d. The optimized standard fortifier, i.e., no breast milk analysis (fortifier 8) lead to adequate growth in about 30% of the feeds. Using target fortification, a higher number of fortified batches met the tolerance range as the number of measurements per week increased. About 40% were inside the growth range when applying one measurement per week. The percentage of appropriately fortified samples increased in increments of ~10% along with the number of measurements per week. The highest accuracy hit the optimized fortifier with one (fortifier 9) or three (fortifier 10) measurements per week of 87–93% (Figure 8).

### Optimization of Macronutrient Content in Native Breast Milk to Reduce the Variation

#### Selection and Combination of Specific Native Breast Milk Samples

Combining samples with opposite extremes [see Methods section Simulation of Selection and Combination of Specific Native Breast Milk Samples (i)] based on one macronutrient (fat, protein, or lactose) or on energy resulted in a decrease in variation of more than 50% for the selected macronutrient, while the other macronutrients showed a smaller decrease in variation. When samples were combined according to energy content, the variation for fat, protein and carbohydrates decreased by 47, 33, and 31%, respectively (Table 5).

**Table 5:** Reduction of variation of fat in native breast milk samples of *n* = 103 subjects, when matching native breast milk batches containing the highest and the lowest macronutrient content, the second highest and the second lowest values, proceeding continuously were combined.

**Matching criterium**	**Variation of macronutrient content in batched native breast milk**			
	**Fat (g/100 mL)**	**Protein (g/100 mL)**	**Lactose (g/100 mL)**	**Energy (kcal/100 mL)**
None	1.5 (0.5, 3.4)	0.6 (0.2, 1.6)	1.3 (0.7, 4.0)	15 (5, 31)
Fat	0.7 (0.5, 1.0)	0.4 (0.3, 0.6)	1.0 (0.8, 1.2)	7.8 (6.2, 10.2)
Protein	1.0 (0.8, 1.4)	0.3 (0.2, 0.5)	0.9 (0.8, 1.2)	10.7 (8.8, 13.9)
Lactose	1.1 (0.8, 1.4)	0.4 (0.3, 0.6)	0.6 (0.5, 0.8)	10.8 (8.2, 14.3)
Energy	0.8 (0.6, 1.0)	0.4 (0.3, 0.6)	0.9 (0.8, 1.2)	7.4 (5.2, 9.9)

*Data show median and interquartiles of ranges Q_0.1_-Q_0.9_ (quantile) (level 2 analysis). Colors highlight the effect of matching on the selected macronutrient compared with native breast milk, yellow - fat, red - protein, green - lactose, blue - energy*.

For the second approach, i.e., pooling breast milk samples with the highest content of either protein or carbohydrates or fat [see Methods section Simulation of Selection and Combination of Specific Native Breast Milk Samples (ii)] increased the selected macronutrient content by 9–15%. However, when ordered by energy or fat, the average macronutrient intake improved for the other macronutrients by 9–13% (Table 6). This approach showed only a minimal reduction in macronutrient variation (data not shown).

**Table 6:** Increase of the macronutrient content in native breast milk samples of *n* = 103 subjects, breast milk samples were selected in groups of 6 or 8 consecutive samples and ordered by either energy, fat, protein, and lactose content.

**Matching criterium**	**Macronutrient content in native breast milk batches**			
	**Fat (g/100 mL)**	**Protein (g/100 mL)**	**Lactose (g/100 mL)**	**Energy (g/100 mL)**
None	3.5 (3.0, 4.1)	1.1 (0.9, 1.3)	6.7 (6.2, 7.2)	63 (57, 70)
Fat	4.1 (3.6, 4.6)	1.2 (1.1, 1.3)	6.6 (6.3, 7.0)	69 (64, 73)
Protein	3.7 (3.3, 4.2)	1.3 (1.1, 1.4)	6.6 (6.3, 6.9)	65 (62, 71)
Lactose	3.6 (3.1, 4.1)	1.1 (1.0, 1.3)	7.0 (6.7, 7.4)	65 (60, 70)
Energy	4.0 (3.6, 4.6)	1.2 (1.1, 1.3)	6.8 (6.4, 7.1)	69 (64, 73)

*Samples containing the three highest values were combined. Values present median and interquartiles (Q_25_, Q_75_) of mean macronutrient of native breast milk (Level 2 analysis). Colors highlight the effect of matching on the selected macronutrient compared with native breast milk, yellow - fat, red - protein, green - lactose, blue - energy*.

#### Simulation of a Milk Bank Setting by Pooling Native Breast Milk

**Figure 9:**
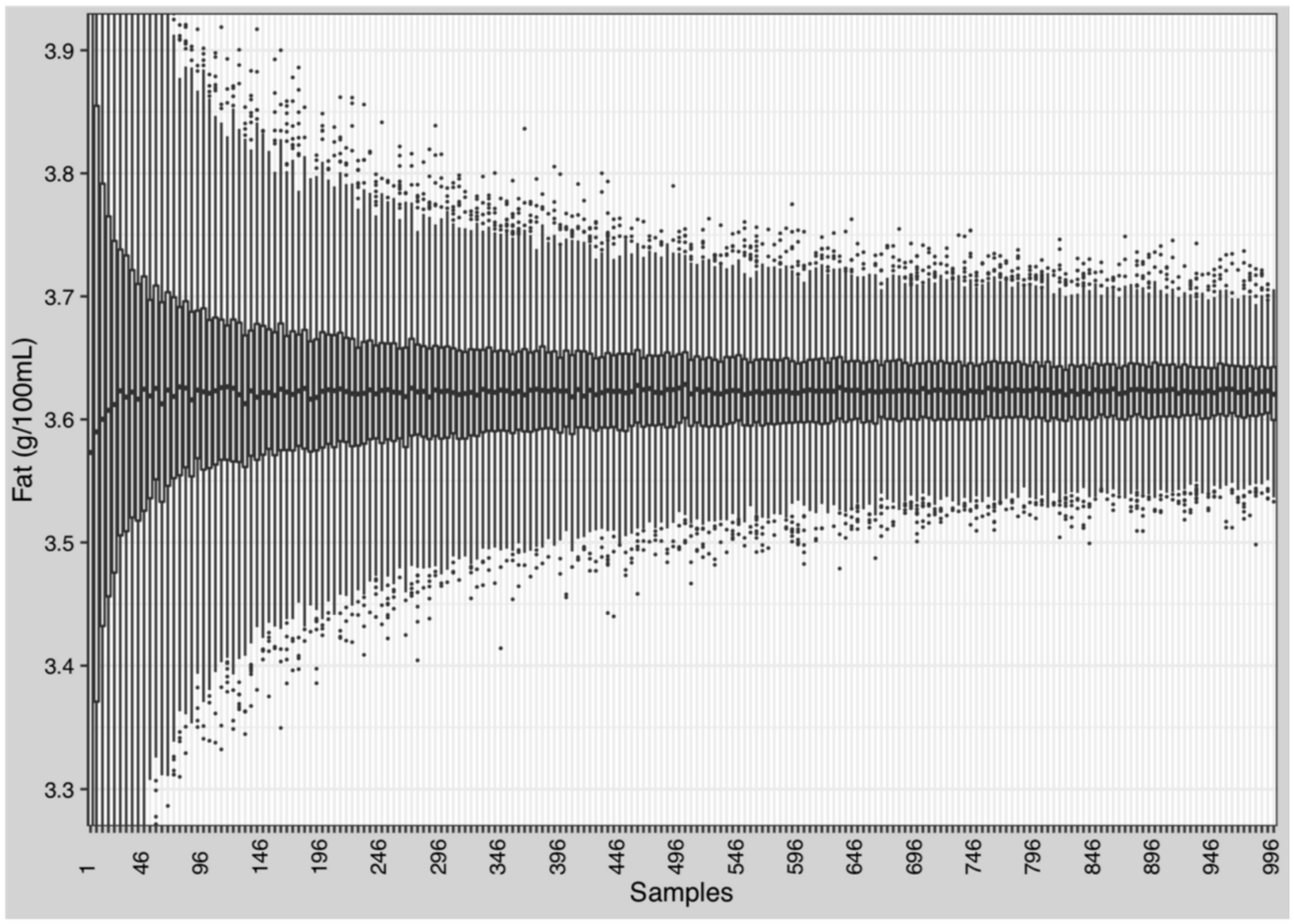
Reduction of the variation of macronutrients and energy in native breast milk. Randomly selection and batching of breast milk samples by stepwise increase from 1 to 996 samples in increments of 5. Each step was repeated 1,000 times and mean and standard deviation were calculated.

Pooling of at least 150 random breast milk samples is needed to reduce the variation of macronutrients to below ±10% (~0.3 g). Figure 9 shows the effects on the variation of fat. Similar effects were observed for protein, carbohydrates, and energy (Supplementary Figure 5).

### Optimizing Macronutrient Content by Partial Lyophilization (Freeze-Drying)

Various factors for partial lyophilization were tested by up-concentrating native breast milk samples to achieve recommended ESPGHAN intakes for macronutrients and energy (Table 7). No lyophilization factor (i.e., hydration factor) could be identified which would achieve recommended levels for all macronutrients in individual batches by using native breast milk. When protein is adjusted to the target level, fat and carbohydrate intakes will be by far too high (energy content of 262 kcal/100 mL). When carbohydrate levels are adjusted to target levels, fat intake will be too high whereas protein intakes would be too low.

**Table 7:** Macronutrient levels in native breast milk (BM) in 150 mL fluid intake.

		**Final macronutrient level**			
	**Lyophilization factor**	Fat (g)^**^	Protein (g)^**^	Carbs (g)^**^	Energy (kcal)^**^
Native breast milk/ESPGHAN	1.0	5.4	1.7	10.1	96
**Target macronutrient per kg/d**					
Fat [6.6 g^*^]	1.2	6.6	2.0	12.3	117
Protein [4.5 g^*^]	2.7	14.7	4.5	27.4	262
Carbs [13.2 g^*^]	1.3	7.1	2.2	13.2	126
Energy 135 [kcal^*^]	1.4	7.6	2.3	14.1	135

*Recommended nutritional intake based on ESPGHAN guidelines reached applying freeze-drying by required lyophilization factor. BM, breast milk; carbs, carbohydrates. A green cell indicates the parameter used for adjustment.*

* *Target intake per kg per day.*

** *Amount per 150 mL breast milk.*

*Blue—values below ESPGHAN targets for macronutrient content, red—values above targets, and green—within targets*.

## Discussion

This study confirms the presence of considerable intra- and inter-individual variation of native breast milk composition with no relationship between macronutrients. This challenges the ability of fortification strategies to achieve recommended intakes. While standard fortification with fixed doses increases the macronutrient content of native breast milk, it does not reduce its variation and the associated risk of unbalanced intakes. Only target fortification, based on at least two breast milk analyses per week in combination with an optimized standard fortifier (fortifiers #9, #10), was able to provide macronutrient intakes that adhered to the recommendations of nutritional committees (20).

Evaluation of milk analysis of 3,338 samples and eight commercially available fortifier shows that standard fortified breast milk meets nutritional requirements as suggested by ESPGHAN in <12% of all feeds. Based on our data, we suggest a “new” optimized standard fortifier in order to meet these recommendations [see chapter Optimized Standard Fortifier (fortifiers #8, #9, and #10)]. The composition of this fortifier differs from current commercially available fortifiers. The optimized fortifier increases the number of appropriately fortified batches; however, 70% of feeds will still have a more or less unbalanced composition of fat, protein, or carbohydrates. In conclusion, standard fortification is not suited to reduce the significant macronutrient variation of breast milk for all preterm infants, and instead transfers the degree of imbalance to higher macronutrient levels (27, 30, 37). It is of interest to note that liquid fortifiers, as opposed to powdered fortifiers, will reduce macronutrient variation: replacement of up to 50% of the volume of breast milk using a fortifier with defined composition will reduce the natural variability to the same degree.

Target fortification reduces the high macronutrient variation of standard fortified breast milk and improves deficient macronutrient content depending on the frequency of native breast milk measurements per week: one measurement per week will allow fortified breast milk to reach the average recommended macronutrient intake, although some intra-individual variation remained. Two or more measurements per week decrease the variation significantly. The best performance was achieved by daily measurements, however, with significantly increased clinical workload. This finding is comparable to our recently published randomized controlled trial, which also suggested to perform two or more weekly breast milk measurements to meet recommended macronutrient intakes and reduce variation (27).

The efficiency of target fortification depends on the composition of the standard product used as base fortifier (Figure 1). Target fortification reduces macronutrient deficiencies in breast milk by adding modular macronutrient products to standard fortified breast milk based on breast milk analysis. However, target fortification cannot reduce a macronutrient that exceeds target levels already after standard fortification as observed for lactose and fat (see Figure 6; lactose: fortifier #1 and #2; fat: fortifier #6, #7, and #8). This has important implications for the nutrient profile of standard fortifiers used in clinical scenarios with target fortification. All commercially available standard fortifiers add between 1 and 1.5 g protein to 100 mL of human milk which rarely leads to protein levels above target leaving room for fine-tuning by adding extra protein. However, their impact on fat, carbohydrate, and eventually energy levels is less consistent because their non-protein composition is quite heterogeneous. Basically, commercially available standard fortifiers can be classified into (i) low-fat/high-carbohydrate, (ii) medium-fat/medium-carbs, or (iii) high-fat/low-carbohydrate containing products. Hence, each group of fortifiers presents a different likelihood for inadequate or excessive intake of macronutrients. Fortifiers with medium-fat/medium-carbs composition provide the most balanced results. In many samples, high-fat/low-carbs fortifiers achieved fat levels above the recommended intake and low carbs levels, whereas low-fat/high-carbs fortifiers provided carbs levels above the recommendations and low-fat intakes (Figure 6).

In this present study, we propose an optimized composition for base fortifiers to reach recommended intakes with feeding volumes of 150 mL/kg/d of milk for most of the batches. The macronutrient composition of these optimized base fortifiers was different if developed for standard (no analysis) or for target (one to three analyses per week) fortification. Neither base fortifier for target fortification should contain fat. Additionally, the composition of the base fortifier for target fortification was determined by the frequency of breast milk analyses. With three measurements per week, the optimized base fortifier had a lower macronutrient content than the fortifier based on one measurement per week. In an individualized approach with more frequent measurements such lower macronutrient levels are needed to prevent overfortification of native breast milk. Conversely, deficient samples will be corrected by individually adding modular single macronutrient products. In an ideal scenario, milk analysis and adjustment by target fortification would occur daily. Hence, the development of a general base fortifier for target fortification would be oriented to adequately fortify samples with higher macronutrient levels (as this is already the case for protein) and thus contain rather low amounts of non-protein components. Besides that base fortifiers would also focus on the important role to provide preterm infants with micronutrients, electrolytes, vitamins, phosphate, and trace elements. The required fine-tuning with macronutrients (fat, protein, carbs), could then individually be made by single macronutrient modulars only according to the breast milk analysis. Consequently, a modern fortification concept for use in clinical routine would aim to standardize intake on the basis of a biologically valuable base liquid with random composition effect by applying real-time component analysis followed by base fortification with (full) micro- and (partial) macronutrients using an optimized base fortifier plus fine-tuning with three modular for protein, fat and carbohydrates.

Our study demonstrated that the commercial liquid fortifier based on human milk provides higher amounts of fat than powdered standard fortifiers. Most of the samples fortified with the liquid fortifier had a higher fat content than recommended by ESPGHAN. However, from a nutritional point of view, the quality of fat from human milk sources cannot directly be compared to fat from non-human milk. The fat fraction of human milk has a particular composition and is constituted by specific fat globules. These fat globules contain glycerophospholipids, sphingolipids, cholesterol, and proteins (36, 38), which may serve as building blocks for cell membranes and cellular structures and also act as bioactive properties supporting the maturation of the gut and the immune system (36). Further, clinical studies have shown that preterm infants fed a human milk diet, containing human milk fat globules, demonstrate body composition, and neurodevelopmental outcomes comparable to term infants (39). While there seem to be benefits of an exclusive human milk diet for preterm infants, there are no studies that have compared the effects of fat derived from human milk to fat from non-human milk on growth and development of preterm infants.

In the present study we were able to show that selective batching of corresponding breast milk batches and/or up-concentrating by partial freeze drying are not appropriate to reduce the variation in the composition of breast milk and to meet the needs of preterm infants. Two main factors limit the effectiveness of these approaches. First, there is only a weak correlation between fat, protein, and carbs (30). Therefore, when breast milk is optimized for one macronutrient, the other two macronutrients may not necessarily reach recommended targets. Secondly, the ratio between macronutrients in native breast milk is different compared to the ratio resulting from adhering to recommendations for preterm infants. In native breast milk, the ratio for protein to fat is 1:3.2 and the ratio for protein to carbohydrates is 1:6.1, while nutritional guidelines suggest a ratio for protein to fat of 1:1.5 and for protein to carbohydrates of 1:2.9. Such differences cannot be overcome by simply up-concentrating batches.

These findings have implications for breast milk preparation. Our data show that on a large scale, like in a milk bank setting, batching of a high number of milk samples (>150) has a high likelihood of creating large volume batches with a similar composition and less macronutrient variation. However, even considering that our sample set might provide a higher variability due to early milk vs. mature milk that a milk bank would have in stock, batch sizes would exceed reasonable numbers. However, on an individual level, mixing, and batching of a small number of breast milk batches only from one mother is unlikely to make a difference of clinical significance. To attain an exclusively human milk diet from mother's own milk by freeze-drying is also infeasible: we were able to demonstrate that the lyophilized human milk concentrates generated in this study fail to provide recommended intakes. The degree of up-concentrating native breast milk to optimize protein intake would lead to a significant over-fortification (more than double) of fat and carbohydrates. Further, freeze-drying could negatively affect infant's intake of vitamins and micronutrients (40) and increase the concentration of environmental pollutants (41, 42).

This study suggests that individualized target fortification of breast milk is able to provide an adequate intake of nutrients at recommended intakes with reduced macronutrient variability. In contrast, current practice of standard fortification provides an uncertain, probably sometimes even unsafe intake of imprecisely defined amounts of macronutrients, unlike the administration of parenteral nutrition or medications which requires accurate dosage and where such practice of random composition would be considered as unacceptable. Unbalanced or excessively high or low intakes of macronutrients may only be recognized by growth failure or metabolic disturbances and hence there inherently is a delay in detection. However, an optimal macronutrient composition is needed to achieve desired body composition outcomes. Recent studies have demonstrated that optimized nutrition leads to higher percentages of fat free mass, which is associated with faster neuronal processing, larger brain size and improved motor development (43–46). Unbalanced macronutrient intake, or under- and over-fortification would result in suboptimal utilization of nutrients and increased risk for adverse long-term health and neurodevelopmental outcomes (33, 47–50).

Strengths of this study include the high number of breast milk batches and mothers (*n* = 3,338 of *n* = 103 subjects), the daily sample collection for a minimum period of 15 days during the NICU stay, and the use of validated methods to precisely measure macronutrient content of breast milk (28–31). Unlike other studies which often present total carbohydrates only (sum of lactose and oligosaccharides), we precisely measured lactose in breast milk using UPLC-MS-MS, allowing us to calculate energy intake without confounding by oligosaccharides. Further, we followed good clinical and laboratory practices guidelines (GCPL) for milk collection, storage, and analysis (28, 51). However, our study also had limitations. Based on the recommendations for macronutrient intake for ELBW infants (birth weight <1,000 g), we studied the achieved nutrient levels using different fortification approaches. However, VLBW infants (i.e., birth weight <1,500 g), intrauterine growth restriction or bronchopulmonary dysplasia may have different nutritional needs, but would be similarly affected by the natural variation of breast milk. Clinical studies are required to explore the effects of fortification approaches on growth for different groups of preterm infants. Another limitation of this study is that we collected milk samples from milk batches which were prepared for 24-h feedings as opposed to collecting samples from individual pumping sessions, which may show greater variation in macronutrient content. Our study did not include data about the quality of macronutrients (human milk protein vs. cow's milk protein), fat (human milk fat globules vs. oil from different sources), and carbs (lactose or glucose polymers). Approaches to reduce the macronutrient variation or improve macronutrient content were performed by theoretical calculation and did not consider the effects of sample preparation and workload. Further, we focused on macronutrients and did not consider micronutrients, vitamins, electrolytes, or trace elements.

## Conclusions

The macronutrient composition of native breast milk varies widely and frequently does not meet the nutritional needs of preterm infants. Batching of native breast milk by using selective strategies as well as concentrating the macronutrients by removing water with freeze-drying is not able to provide the recommended macronutrient intake to all preterm infants. Most commercial standard fortifiers do not achieve ESPGHAN recommended intakes. An optimized fortifier for standard fortification could only reach recommended intake in 30% of feeds. Target fortification reduces the inter- and intraindividual macronutrient variation and can achieve recommended ESPGHAN intake for individual infants. However, the composition of the base fortifier needs to be adjusted for best practice target fortification and is different from a fortifier originally developed for standard fortification. More frequent breast milk measurements and recipe refinement for fortifiers (and modular) to be used in target fortification would improve the macronutrient intake and thus growth.

## Data Availability Statement

The datasets presented in this article are not readily available because restricted by the Study protocol and Research Ethics Board of McMaster University. Requests to access the datasets should be directed to kinderzentrum@klinikum-nuernberg.de.

## Ethics Statement

The studies involving human participants were reviewed and approved by Research Ethics Board of McMaster University. Written informed consent to participate in this study was provided by the participants' legal guardian/next of kin.

## Author Contributions

CF and NR: conceptualization, methodology. HS, MR, and NR: formal analysis. GF and SF: milk analysis. GF, NR, and SF: data curation. EY, NR, and SF: writing—original draft preparation. CF, MR, NR, and SF: visualization. NR: supervision. CF: project administration. All authors writing—review and editing. All authors have read and agreed to the published version of the manuscript.

## Funding

This study was partially funded by Canadian Institute of Health Research, #MOP-125883.

## Conflict of Interest

The authors declare that the research was conducted in the absence of any commercial or financial relationships that could be construed as a potential conflict of interest.

## Publisher's Note

All claims expressed in this article are solely those of the authors and do not necessarily represent those of their affiliated organizations, or those of the publisher, the editors and the reviewers. Any product that may be evaluated in this article, or claim that may be made by its manufacturer, is not guaranteed or endorsed by the publisher.
