# Supplementary material for: Individualized Target Fortification of Breast Milk: Optimizing Macronutrient Content Using Different Fortifiers and Approaches
Source: Front Nutr. 2021 Sep 21;8:652641. doi: 10.3389/fnut.2021.652641 (PMC8490648; doi:10.3389/fnut.2021.652641)
Supplement: Supplementary file 1 [file Data_Sheet_1.docx]

***Supplementary Material***

**Native Breast milk**

**Supplementary Table 1:** Macronutrient content in native breast milk n=3,338 samples from n=103 subjects (Level 1 analysis)

| All breast milk samples | | | |
| --- | --- | --- | --- |
|  | Mean ± SD | Median (Q_0.25_, Q_0.75_) | Quantile Q_0.1_, Q_0.9_ |
| Fat (g/100 mL) | 3.6 ± 0.9 | 3.5 (3.0, 4.1) | 2.6, 4.8 |
| Protein (g/100 mL) | 1.1 ± 0.3 | 1.1 (0.9, 1.3) | 0.8, 1.6 |
| Carbs (g/100 mL) | 6.7 ± 0.8 | 6.7 (6.2, 7.2) | 5.8, 7.7 |
| Energy (kcal/100 mL) | 64 ± 9 | 63 (57, 70) | 53, 76 |
| Protein:Energy (g/100 kcal) | 1.8 ± 0.4 | 1.7 (1.5, 2.1) | 1.3, 2.4 |
| Carbs/NPE (%) | 46 ± 7 | 46 (41, 50) | 37, 54 |

**Supplementary Table** 2: Distribution of individual mean intake, individual IQR, and individual quantile distances (Level 2 analysis)

| Individual variation, median (min, max) | | | |
| --- | --- | --- | --- |
|  | Mean intake | IQR Q_0,25_-Q_0,75_ | Quantile distance Q_0.1_ - Q_0.9_ |
| Fat (g/100 mL) | 3.6 (2.2, 5) | 0.8 (0.3, 1.9) | 1.5 (0.5, 3.4) |
| Protein (g/100 mL) | 1.1 (0.8, 1.8) | 0.3 (0.1, 1.1) | 0.6 (0.2, 1.6) |
| Carbs (g/100 mL) | 6.6 (5.7, 7.8) | 0.7 (0.3, 1.9) | 1.3 (0.7, 4.0) |
| Energy (kcal/100 mL) | 64 (50, 78) | 8 (2, 18) | 15 (5, 31) |
| Protein:Energy (g/100 kcal) | 0.4 (0.2, 1.0) | 0.8 (0.4, 1.6) | 1.8 (1.3, 2.9) |
| Carbs/NPE (%) | 46 (37, 58) | 6 (3, 13) | 12 (6, 26) |

**Protein per energy ratio in standard fortified and target fortified breast milk**


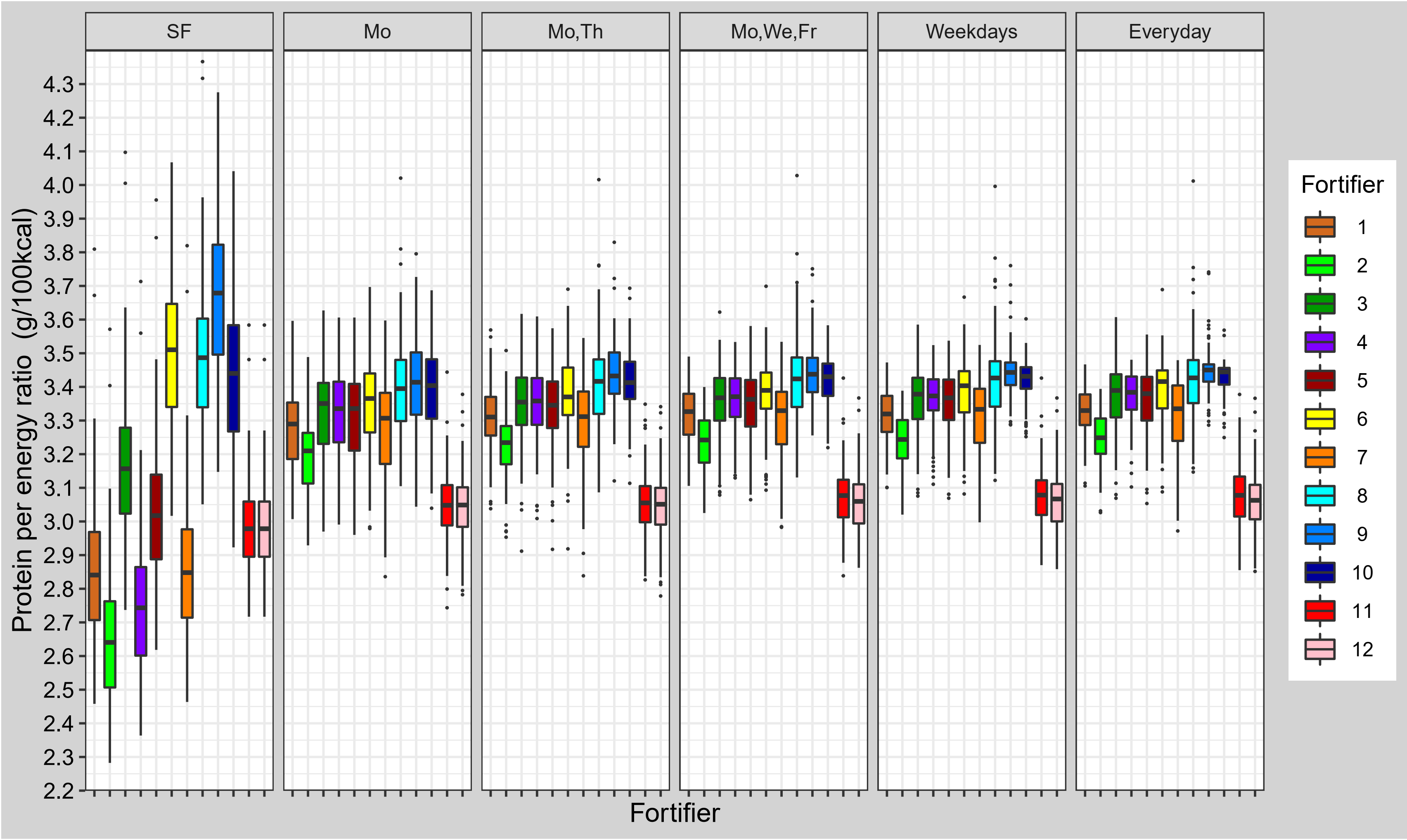


**Supplementary Figure 1:** Protein per energy ratio in standard and target fortified breast milk using 12 fortifiers. Data include 103 subjects in a total of 3,338 breast milk samples.


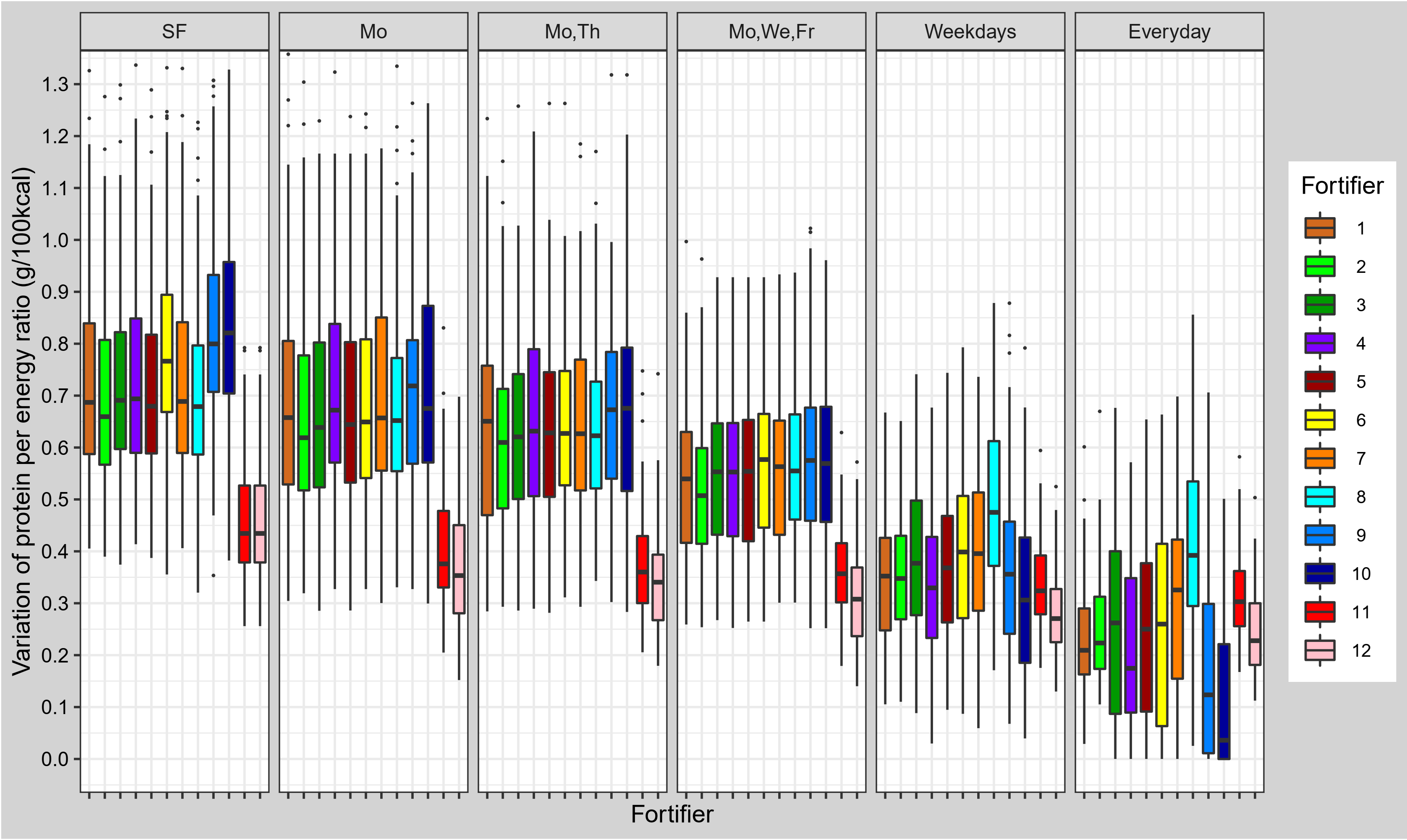


**Supplementary Figure 2:** Variation of protein per energy ratio, boxplots show distribution of the quantile distances (Q_0.1_ to Q_0.9_) of n=103 subjects in fortified breast milk using 12 fortifiers in different approaches.

**Carbs energy per non-protein energy**


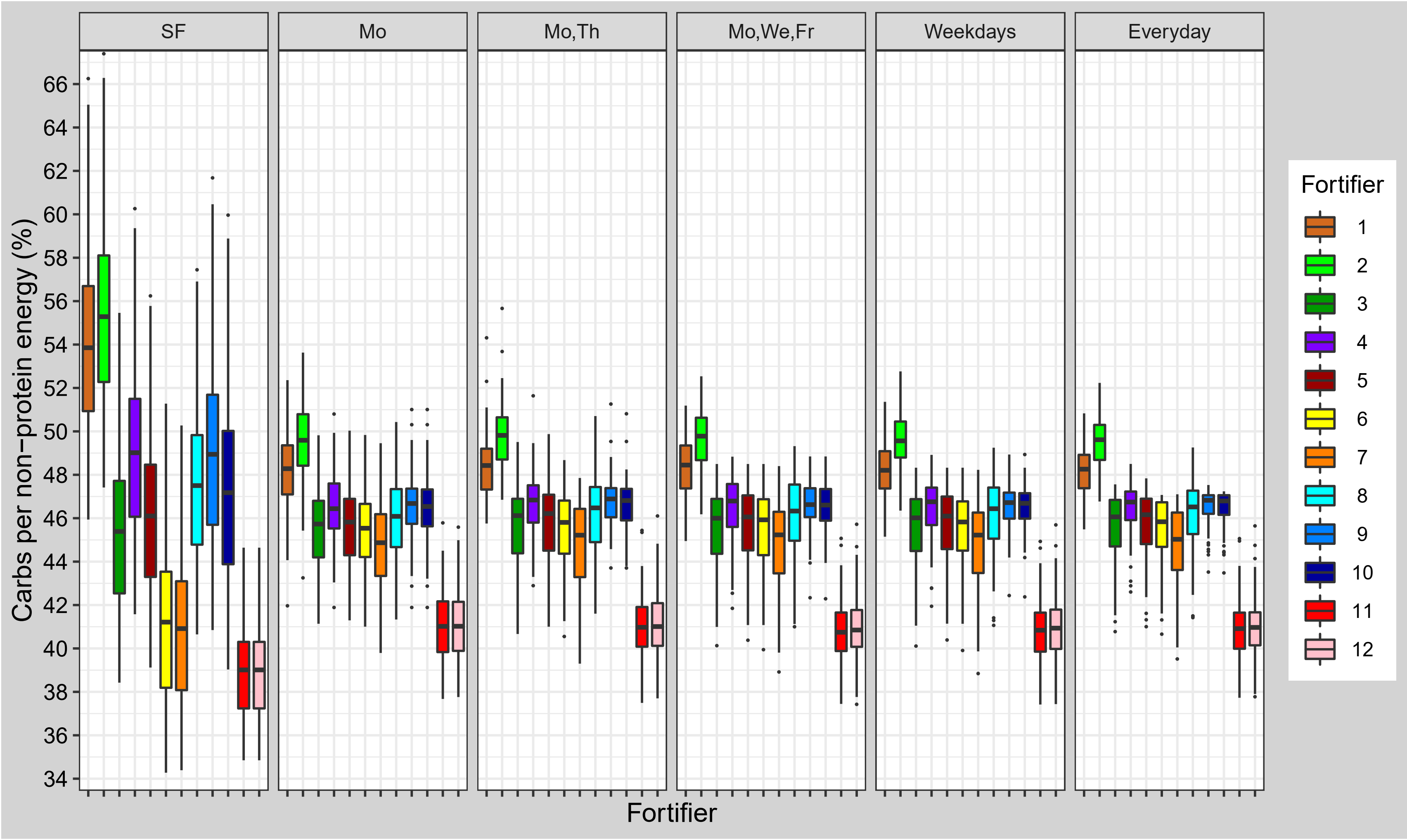


**Supplementary Figure 3:** Carbs energy per non-protein energy in standard and target fortified breast milk using 12 fortifiers. Data include 103 subjects in a total of 3,338 breast milk samples.


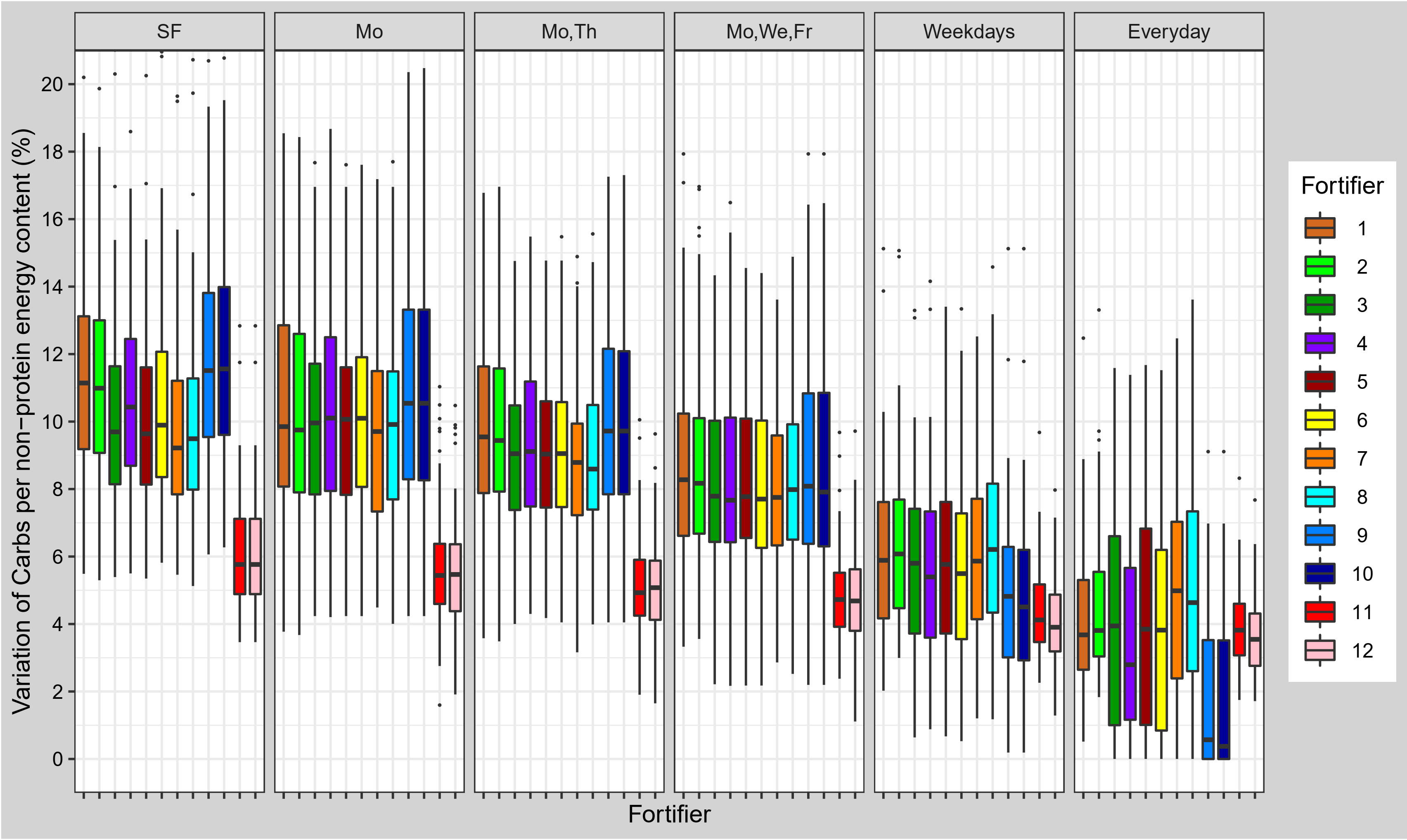
 **Supplementary Figure 4:** Variation of carbs energy per non-protein energy, boxplots show distribution of the quantile distances (Q_0.1_ to Q_0.9_) of n=103 subjects in fortified breast milk using 12 fortifiers in different approaches.

**Batching of breast milk to reduce the macronutrient variation: Model of scenarios for a milk bank**


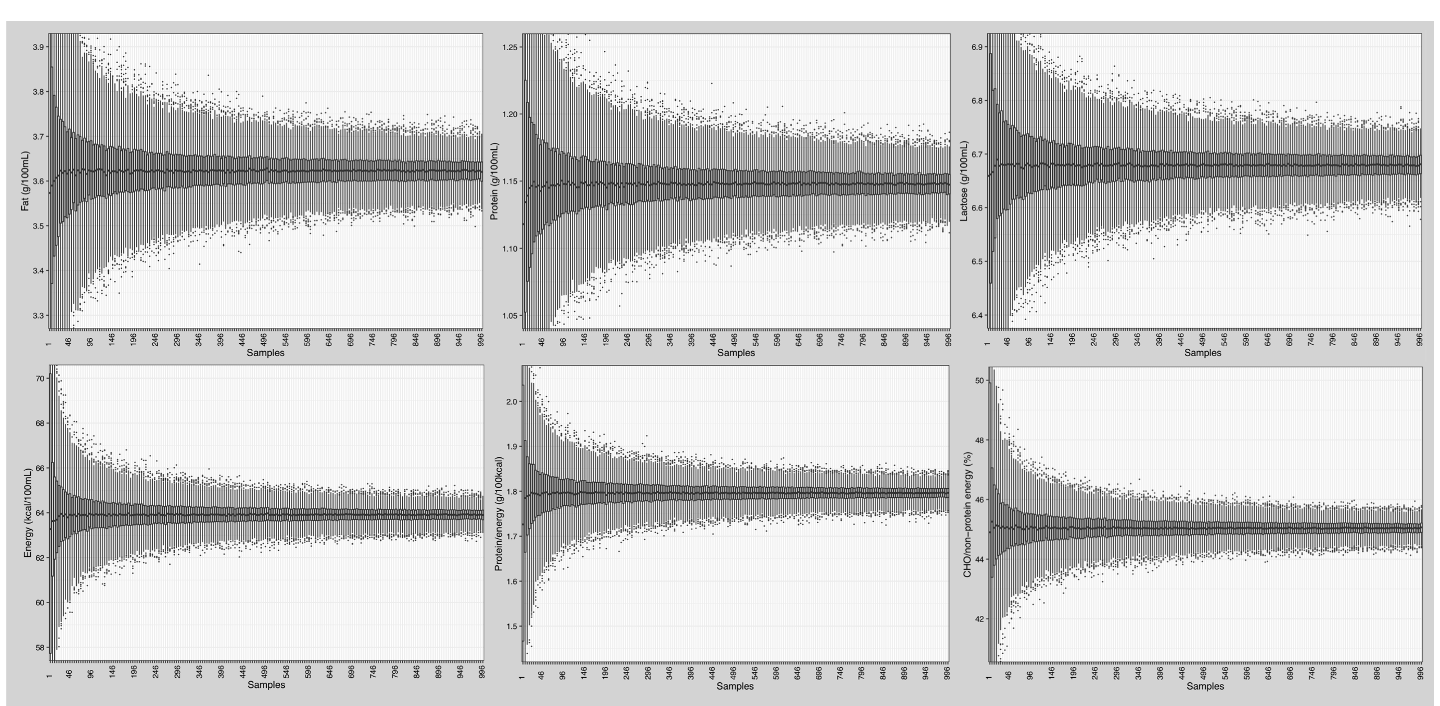


**Supplementary Figure 5:** Reduction of the variation of macronutrients and energy in native breast milk. Randomly selection and batching of breast milk samples by stepwise increase from 1 to 1,000 samples in increments of 5. Each step was repeated 1,000times and mean and standard deviation calculated.
